# Performance of Repaired Concrete under Cyclic Flexural Loading

**DOI:** 10.3390/ma14061363

**Published:** 2021-03-11

**Authors:** Boyu Wang, Rishi Gupta

**Affiliations:** Department of Civil Engineering, University of Victoria, 3800 Finnerty Road, Victoria, BC V8W 2Y2, Canada; boyuwang@uvic.ca

**Keywords:** cyclic loading, repair materials, bond, hysteric behavior

## Abstract

There is limited research reported on the effect of cyclic loading on cement-based repair materials as conducting such tests is time consuming. To overcome this issue, this study utilized a novel loading regime consisting of cycle groups with increasing stress amplitude to accelerate the test process. The Palmgren-Minder rule was used to estimate the fatigue life of repaired specimens. Specimens repaired with Mix M (cementitious repair mortar), which was estimated to have the highest 2-million-cycle fatigue endurance limit (77.4%), showed the longest fatigue life (95,991 cycles) during the cyclic loading test, the highest slant, and splitting bond strength among all repair mixes. The estimated two-million cycle fatigue endurance limit of Mix S (70.8%) was very similar to that was reported in literature (71%) using the traditional loading method. This study confirms the usefulness of Palmgren-Minder rule on estimating the fatigue life of repaired specimens. Additionally, the use of the novel loading regime showed the benefit of shortening the test process while producing results similar to those from using traditional loading methods. To improve the prediction accuracy, future research is required to modify the failure criteria to accommodate specimens that may not fail even when the average flexural strength is met.

## 1. Introduction

Concrete is widely used as a traditional building material in construction engineering, road engineering, and bridge engineering due to its low cost and excellent mechanical and durability performance [1]. However, due to external loading, environmental temperature changes, and ingress of aggressive substances, concrete can suffer from cracking and spalling, which substantially compromises the durability and safety of concrete structures [2]. It is a common practice to use repair materials to restore and extend the service life of concrete structures [3,4]. Most of the repair materials can be classified into three categories including polymer-modified cement-based materials, polymer materials, and cement-based materials [5]. Conventional Portland cement-based material is one of the most widely used materials for concrete repair [6], but has issues such as high drying shrinkage [7], permeability [7], and susceptibility to aggressive chemicals [8]. Polymer-modified cement repair materials, on the other hand, have advantages such as superior chemical resistance [9], low cure shrinkage [6], and good adhesion characteristics [10]. Some of the commonly used polymers include polyvinyl acetates, styrene butadine rubber, and polyvinyl dichlorides, which serve as a water reducing plasticizer that improves the workability while lowering the shrinkage.

In the recent decade, the fatigue behavior of construction materials received great attention because natural or human activity-induced loads on buildings are often cyclic in essence [11]. When concrete is subjected to cyclic loading, there will be residual strain accumulating after each cycle, which indicates the internal progressive permanent structural change in concrete [12]. The fundamental mechanisms behind fatigue failure can be explained using the stress concentration and fracture mechanics [13], and various approaches have been used by researchers to predict the concrete failure due to fatigue loading. One of the widely accepted approaches is based on empirically derived *S-N* (Stress-Number) diagrams, where *S* represents the load during cyclic loading and *N* means the number of cycles required to cause failure. Unlike most metals, concrete does not have a fatigue limit which means it will eventually fail after certain loading cycles. An example is that plain concrete subjected to repeated uniaxial tensile stresses appeared to fail before *N* reaches 2 × 10^6^ cycles regardless of the stress level [14]. To quantify the fatigue damage of materials, different damage variables are introduced, and they are based on fracture mechanics [15,16,17], numerical approach [18,19], or continuum damage mechanics [20,21,22]. The fracture mechanics method estimates fatigue crack propagation by measuring the stress intensity factor which indicates the stress state of the materials. However, the analysis of results can be quite cumbersome if the nonlinear mechanistic models are used [23]. Additionally, the complex detection equipment is indispensable for real-time monitoring of crack development [24]. The numerical approach involves using finite element method to simulate the crack growth of materials under fatigue loading [25]. The continuum damage mechanics considers the creation and growth of microcracks, which is discontinuous in its nature, to be continuous at a larger scale [26]. Damage leads to the change of materials properties such as elastic modulus, hardness, density, etc. Therefore, some commonly used variables that are chosen to quantify damage include elastic modulus, maximum strain, residual strain, energy dissipation, and ultrasonic pulse velocity [26]. The advantages of using fatigue damage variables based on continuum damage mechanics include: (1) easy measurement and application in engineering practice; (2) distinct physical meaning; (3) taking initial damage during fatigue loading into account. As a result, this study employs the method of continuum damage mechanics to derive *S-N* diagrams and estimate the fatigue life of materials under fatigue loading. In 1945, Miner [27] introduced the concept of damage accumulation in a simple form. This rule assumes linear accumulation of fatigue damage with the increasing number of cyclic loads. Over the years, efforts have been made to formulate this rule involving more factors such as fatigue loading history [28], stress ratio [29], thermodynamics potential [30], and fatigue inelastic parameters [20]. The modified Palmgren-Miner’s rules have improved accuracy in predicting fatigue life, but face challenges in being widely used in current building codes due to their complex forms [31].

In the case of repaired structures, it is important to ensure sufficient fatigue life for the bond between the parent concrete and repair materials. Shah et al. [32] studied the fracture behavior at a concrete-concrete interface under fatigue loading conditions. It was found that the fatigue life of intact specimens was the highest and decreased with increasing difference between the elastic moduli of materials on either side of the interface. Shah et al. [33,34] reported similar findings that the mismatch in elastic modulus and in compressive strength between the parent and repair materials increases the vulnerability to cracking when a patch repair system is under quasi-static loading conditions. Ong et al. [35] investigated the fatigue behavior of concrete beams repaired with steel-fiber cement-based mortars. They found that repair concrete beams survived 100,000 load cycles without any delamination and significant loss in stiffness if the maximum amplitude of cyclic loading was below 45% of ultimate static strength. Some researchers [36,37] investigated the steel reinforced concrete beams retrofitted with ultra-high performance concrete. They used epoxy-based adhesives to bond repair concrete plate with the substrate concrete and tested them under fatigue loading. It is found that the fatigue life of repaired beams was longer than the control (intact) beam [36].

In summary, most of the aforementioned literature focused on the bond strength of cement-based repair concrete under fatigue loading, but scarce work studied other types of repair such as polymer-modified cementitious mortar, which outperform cement-based concrete repair in many aspects [9,38,39,40]. Additionally, apart from the exhaustive studies [20,28,29,30,31,41] that used Palmgren-Miner’s rule and its modified forms on concrete materials, there are insufficient studies about the applicability of Palmgren-Miner’s rule on predicting the fatigue life of repaired structures. In this study, authors aim to determine the applicability of Palmgren-Miner’s rule to estimate the fatigue life of repaired concrete structures. Since cyclic loading is time-consuming, a modified loading regime consisting of cycle groups of increasing cyclic stress amplitude is used to accelerate the test process. A method based on fatigue stress to flexural strength ratios is also proposed to derive the *S-N* curve. The estimated fatigue life in this study is compared with the results in previous literature to determine its liability. Additionally, the failure mode, hysteretic behavior, and dynamic elastic modulus drop of repaired samples after cyclic loading are analyzed and discussed, which is used to validate the predicted *S-N* curves. The findings of this study not only provide information on how to conduct a rapid and easy estimation of the fatigue life of repaired structures but explore the fatigue resilience performance of various repair materials.

## 2. Materials and Methods

**Figure 1:**
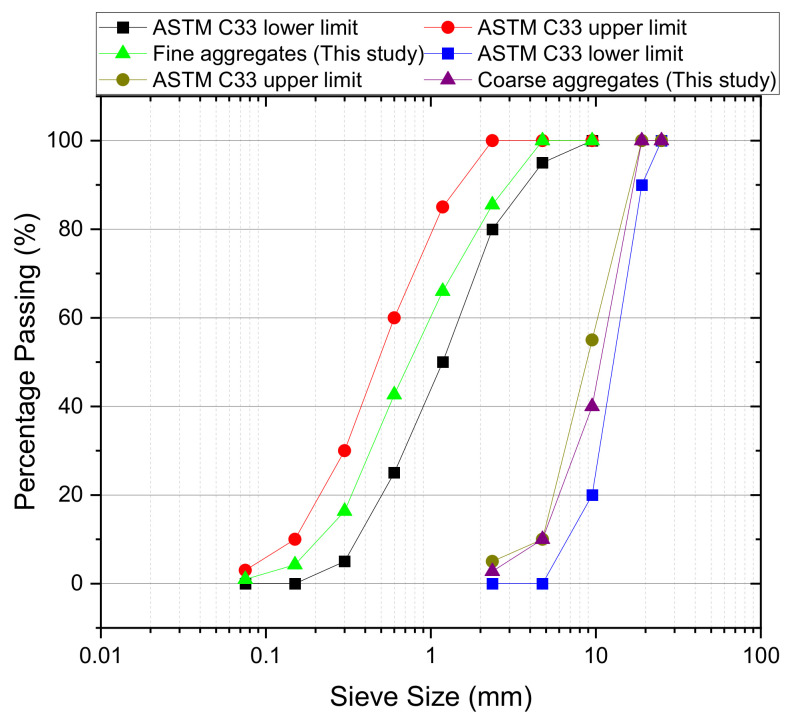
Gradation curves of aggregates used in this study (Redrawn from [42]).

A total of 4 mixes were prepared including one control mix developed in the lab and three commercial repair products locally available in the market. The control mix for the substrate, named Mix S, received the repair and had a design compressive strength of 50 MPa. The rationale for using 50 MPa concrete as the substrate was to minimize its own degradation (prior to that of the applied repair material) due to external loadings such that the fatigue resilience performance of different repair materials could be determined. Additionally, the use of Mix S as the substrate is the same as a previous study by authors [42] and its use provides for better research continuity. Table 1 summarizes the mix design details of Mix S, and the gradation information of aggregates is shown in Figure 1. Based on the gradation curves, the fineness moduli of coarse and fine aggregates were calculated to be 6.48 and 2.85 as per ASTM C136 [43]. The water to material ratio (w/m) for Mix S, F, P, M were 0.067, 0.1, 0.18, and 0.09, respectively. In this study, cementitious repair mortar (Mix M), cementitious repair concrete (Mix F), and polymer-modified cementitious mortar (Mix P) are used. These three types cover most of the commercial repair products on the market and thus are representative. To have sufficient workability, 2.37 kg, 4.26 kg, and 2.5 kg of water were added to Mix M, Mix P, and Mix F per bag, respectively, as recommended by the material manufacturers. The slump of Mix S was adjusted to 60 mm which meets the range stipulated by ACI 211.1 [44]. Manufactures provided the setting time information which is shown in Table 2. The fresh properties of the repair and control mixes include air content and slump which are measured following ASTM C231 [45] and ASTM C 143 [46] respectively. The hardened properties include compressive strength ($f_{c}^{\prime }$) and density at 28 days which were determined following ASTM C39 [47]. All this information can be found in Table 2. More information about the properties such as freeze–thaw and corrosion resistance performance of the repair materials can be found in Wang et al. [42,48,49] and Bajaj et al. [50].

### 2.1. Specimens

#### 2.1.1. Prisms

**Figure 2:**
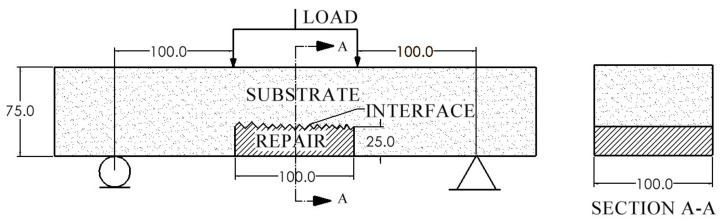
Prismatic specimens under four-point loading (all dimensions are in mm).

In field applications, a common practice to rehabilitate structures is to remove delaminated concrete, clean the parent substrate and rebars, and apply the repair materials. In order to simulate in situ-damaged structures, prismatic beams with an induced cut on the tension side were prepared. Notched beams were prepared using Mix S and received the repair materials (Mix M, F, and P). All specimens were cast with a dimension of 75 mm × 100 mm × 400 mm, as shown in Figure 2. For comparison purposes, specimens made of Mix S with no cut were prepared as well. Additionally, the material supplier of Mix F specifies the minimum depth of repair to be 25 mm which is the upper limit prescribed by the supplier of Mix M. Given these constraints, all specimens in this study have a repair depth of 25 mm. The shape of the repair is selected to be square (100mm × 100 mm) in order to prevent problems such as feather edge. Experience has shown that repair areas with feather (very thin) edges usually fail quickly.

**Figure 3:**
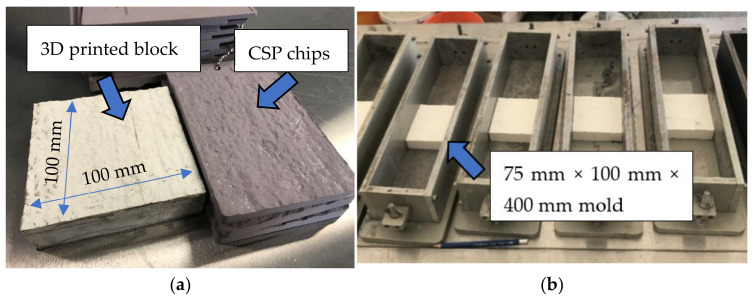
Mold preparations (**a**) 3D printed block and #6 CSP chip (**b**) 3D printed blocks in molds.

Prior to the application of the repair, surface roughening is an important step to ensure a good bond. Typically, quantifying the roughness of a concrete surface is mainly through comparing the target surface with nine standard concrete surface profile chips. These chips were proposed by International Concrete Repair Institute (ICRI) [51]. In this study, surface profile chip #6 was selected as the target surface profile on the surface receiving repair on the substrate as shown in Figure 3a. This was recommended as the typical target profile by the industry partner who has many years of experience with repair and rehabilitation of concrete structures. In order to achieve a good consistency between specimens in terms of surface roughness, an innovative plastic block with certain surface roughness was conceived and designed by the authors. As shown in Figure 3a, the block was made by 3D printing on a plastic base following a model that is produced by 3D scanning the #6 surface profile. A high-resolution 3D printer named Ultimaker 3 was used to print the inserted blocks. This 3D printer has up to 20 micro resolution which ensures printing objects with high accuracy.

The block was introduced into the mold before concrete placement. Figure 3b shows the 3D printed block placed at the bottom of the mold. Concrete was placed into the molds and placed on a vibrating table for a ten-second consolidation. Specimens were cured in ambient conditions at 18 ± 2 °C for 24 h before the inserted blocks were removed. Three types of repair materials (Mix M, F, and P) were applied to the cavity. Another 24 h later, all specimens were demolded and transferred to the water bath for water curing. The range of water curing temperature was 23 ± 2 °C Prior to testing, all specimens were cured in water for 28 days.

#### 2.1.2. Cylinders

**Figure 4:**
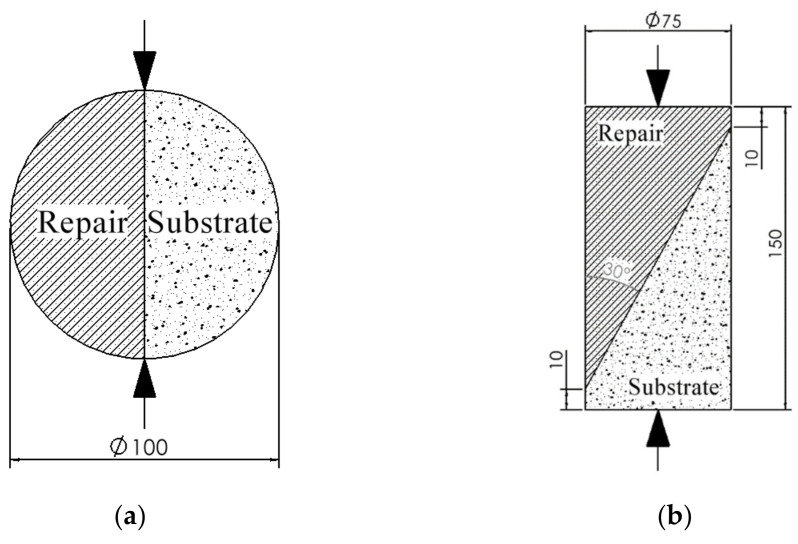
Bond test sketches (**a**) Splitting tensile test (**b**) Slant shear test (all dimensions in mm).

Cylindrical specimens were prepared for splitting tensile and slant shear bond tests. The dimension of the prepared specimens is illustrated in Figure 4. Cylinders of dimensions 100 mm×200 mm and 75 mm ×150 mm were first cast and cured in water for over 28 days at 23 ± 2 °C before receiving the repair. The 75 mm ×150 mm cylinders were saw cut at an angle of 30° for splitting tensile test following ASTM C882 [52]. The 100 mm×200 mm cylinders were saw cut along diametrical lines for slant shear test. They were then repaired with Mix F, M, and P, and kept in water at 23 ± 2 °C for 14 and 28 days before the bond tests were performed.

### 2.2. Flexural Strength & Cyclic Loading Test

**Figure 5:**
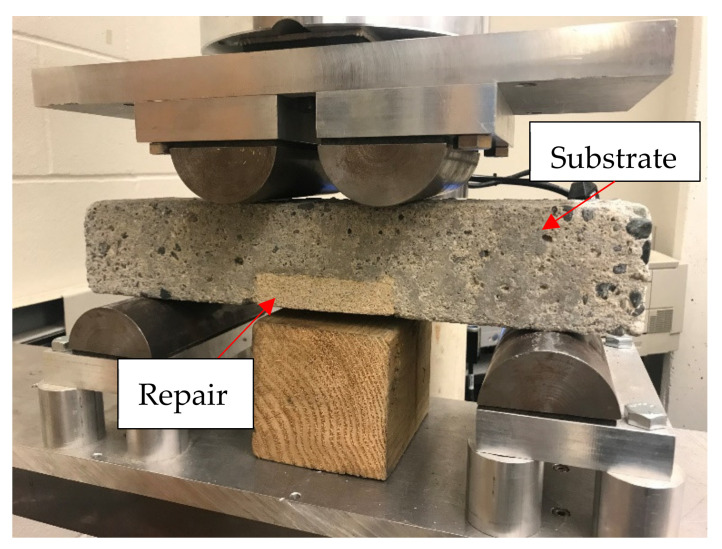
Test setup for cyclic loading.

A third-point loading test was conducted to determine the static flexural strength of the repaired prisms as per ASTM C78 [42]. An MTS 810 machine with 250 kN load capacity was used to load the specimens. A total of 3 specimens for each mix were tested and the modulus of rupture was calculated using Equation (1). The specimens were loaded with the repair materials on the tension side. According to ASTM C78, the loading rate was set as 2323 N/min such that the maximum stress on the tension surface is 1.2 MPa/min. The loading setup is shown in Figure 5.

(1) $$\sigma_{u}=\frac{PL}{bd^{2}}$$

where $\sigma_{u}$ is the ultimate stress of the material, *P* is the maximum applied load indicated by the test machine, *L* is the span length, *b* is the average width of specimen, and *d* is the average depth of specimen.

Following the flexural strength test, a series of specimens were exposed to flexural cyclic loading, and the test setup was the same as shown in Figure 5. Conventional cyclic loading may consume a large amount of time and have risks of failing the specimens in the first few cycles especially when the applied stress is close to the ultimate strength. In order to overcome these problems, all specimens in this work were subjected to cycle groups of increasing amplitude, and a new approach was used to obtain the *S-N* curve for repaired specimens. In this study, the load variation was sinusoidal with a frequency of 10 Hz. Different loading force groups were used on intact and repaired beams as they had different flexural strength. Table 3 shows the details of loading protocols adopted in this study. The moduli of rupture of repaired beams with different mixes were first determined, which provides the benchmark for setting cyclic loading parameters. In the case of intact beams (Mix S) that was cured for 28 days, the first 10,000 cycles exert a maximum force of 4235 N, which corresponds to 55% of the averaged modulus of rupture of Mix S. The minimum cyclic force was set as 800 N for all the specimens. If the specimen has not failed after the first cycle group, the maximum cyclic load will increase to 5005 N, which corresponds to 65% of the averaged modulus of rupture value. The loading forces increase at an interval, which corresponds to approximately 10% of the modulus of rupture value. The cyclic test continues until the resulting stress reaches the average modulus of rupture value or until the specimen fails, whichever happens first. Some specimens may not fail even when the maximum cyclic load has reached 100% of the average ultimate load. In this case, the cyclic test continues with increased cyclic loading force (200 N above the average ultimate load) and loads the specimen for another 10,000 cycles. The loading force keeps increasing until the specimen breaks. The number of cycles at failure is recorded.

In this study, the use of cycle groups to obtain an *S-N* curve was adopted from the work of Nieto et al. [53]. Different than [53], this study used cycle groups to test repaired concrete beams. The determination of *S-N* curve was based on the assumption that the curve can be approximated by a straight line in a logarithmic scale, as shown in Equation (2). By means of Palmgren-Miner rule and Goodman linear model [53], Equation (2) can be further derived into Equation (3), which can be used to calculate the straight line slope *b* in Equation (2). As demonstrated in Equation (3), the accumulative damage factor $D_{i}$ will reach 100% when the specimen fails.

(2) $$S=\sigma_{u}N^{b}$$

where *N* is the number of cycles to failure, *S* is the stress applied on the specimen, and *b* is the slope of the straight line.

(3) $$D_{i}=\overset{i=k}{\underset{i=1}{\sum }}n_{i}{\left(\frac{\sigma_{u}-\sigma_{i}^{m}}{\sigma_{i}^{a}}\right)}^{1/b}$$

where $D_{i}$ is the accumulative damage factor expressed in percentages, $n_{i}$ is the number of cycles applied at *i*th cycle group, $\sigma_{i}^{m}$ indicates the mean stress value at *i*th cycle group, and $\sigma_{i}^{a}$ is the alternating stress value at *i*th cycle group.

### 2.3. Non-Destructive Test (NDT) Method

**Figure 6:**
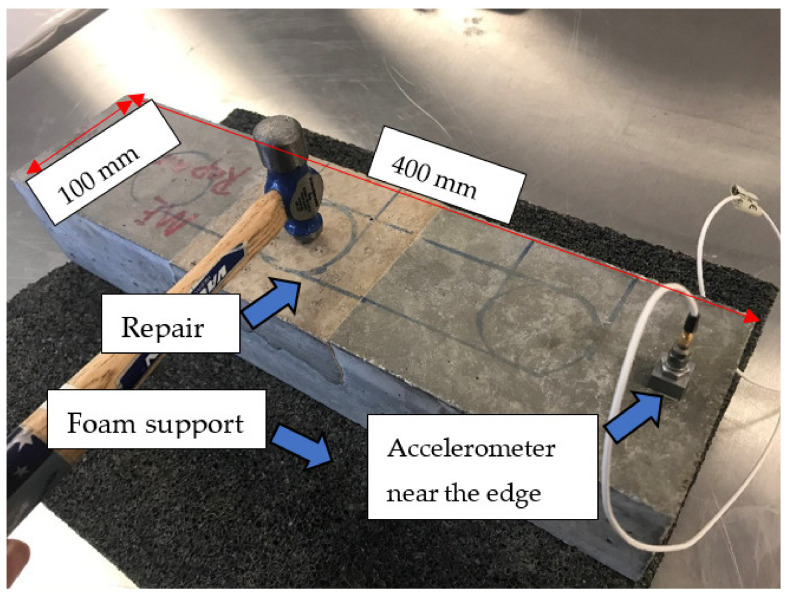
Resonant frequency test setup.

The Resonant frequency test was performed when each cycle group is completed to determine the changes in dynamic modulus of elasticity ($E_{dyn}$). According to ASTM C215 [41], the resonant frequency and $E_{dyn}$ can be determined using an accelerometer. In this study, the resonant frequency tester consisted of a PCB Piezotronics 353B15 accelerometer and a data acquisition system manufactured by Olson Instruments. The transverse resonant frequency of the 1st mode was used to calculate $E_{dyn}$. The specimens were first placed on a foam piece to allow for free vibration. An accelerometer was then attached to the end of the specimen through an adhesive grease. A hammer was used to strike the middle of the specimen, and the vibration signal was captured by the accelerometer. The test setup is shown in Figure 6.

### 2.4. Bond Test Methods

The interfacial tensile and shear bond strength between substrate concrete (Mix S) and repair materials are determined using methods shown in Figure 4a,b. The splitting tensile test follows the standard procedures stipulated by ASTM C496 [54]. This test applies a diametral compressive force along the concrete-repair interface at a rate of 0.5 kN/s, and the peak load at failure was recorded. Equation (4) is used to calculate splitting tensile bond strength ($\sigma_{t}$).

(4) $$\sigma_{t}=\frac{2P}{\pi LD}$$

where *P* is the load at failure, *L* and *D* are cylinder length and diameter.

The slant shear test was performed following ASTM C882 [52]. The specimen was loaded at a rate of 1.1 kN/s until failure as shown in Figure 4b. Equation (5) is used to calculate slant shear bond strength (τ).

(5) $$\tau =\frac{1}{2}\sigma_{0}\sin2\alpha$$

where τ is slant shear bond strength, $\sigma_{0}$ is the vertical stress applied at the cylinder edge, α is the bond plane inclination angle which is $30^{{}^{\circ}}$ in this study. FORNEY compression testing machine (model: F-650), which has a load capacity of 2891 kN, was used for this test.

## 3. Results and Discussions

### 3.1. Bond Strength

**Figure 7:**
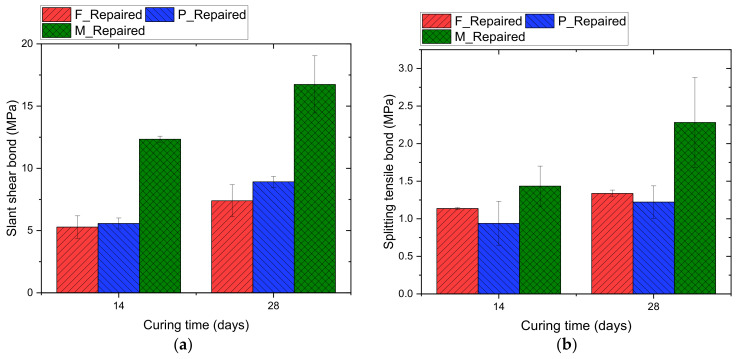
Bond stresses at failure for different repair materials (**a**) slant shear bond (**b**) Splitting tensile bond.

The life cycle of the repaired beams under cyclic load is affected by the bond strength of the repair materials [55]. In this study, the shear and tensile bond strength of three repair materials are determined, and the average results of three specimens from each mix are shown in Figure 7. Among all repair mixes, Mix M (cementitious repair mortar) shows the best bond performance with an average slant shear bond strength of 12.3 MPa and 16.7 MPa and tensile bond strength of 1.4 MPa and 2.3 MPa at 14 and 28 days of curing, respectively. Past research reported that the slant shear bond strength of different repair materials ranged from 5 MPa to 70 MPa [56,57,58,59,60], and the splitting tensile strength ranged from 0.5 MPa to 5 MPa [57,58,60,61,62]. The large deviation in bond test results could be due to many reasons [63]. The factors that affect bond strength include surface roughness, cleanliness, soundness [64], chemical adhesion and cohesion [65], mix design, and curing regimes of the repair materials [66]. Some recent research [57,67] reported that the bond strength of ultra-high-performance concrete (UHPC) used as repair materials is excellent. The main reason is the dense interfacial transition zone between UHPC and the substrate as UHPC has a very low water-to-cement ratio [58]. Additionally, the absence of coarse aggregate in UHPC contributes to better compaction during concrete placement. In this study, Mix M (cementitious repair mortar) possesses the highest strength which potentially results in a better bond compared to other repair mixes. Additionally, since the repaired samples in this study have the same roughness (because of the identical 3D printed blocks used for surface preparation) and curing regimes, the dominant factor is attributed to the mix design. Past research has shown that the involvement of fibers [68,69] and polymers in the mix [70] is beneficial in improving the adhesion strength of the repair materials. In this study, the polymer-modified cementitious mortar (Mix P) contains polypropylene fibers with an approximate dosage of 0.2% by weight of all the constituents in the mix. Though the compressive strength of Mix P is 10 MPa less than Mix F (cementitious repair concrete), Mix P exhibited similar slant and tensile bond strength compared to Mix F. In Figure 7b, mix M and P samples have relatively large variations compared to mix F samples. This could be attributed to the bond test method used in this study. This is supported by the past literature [59,71] that has shown that the scatter of bond test results using various test methods could be large. It is observed that some specimens of Mix M and P samples experienced slight crumbling along with adhesion failure during the bond test, which could result in variations of bond test results. Additionally, in a study by Feng et al. [58], the scatter in bond test results varies with different mixture designs.

**Figure 8:**
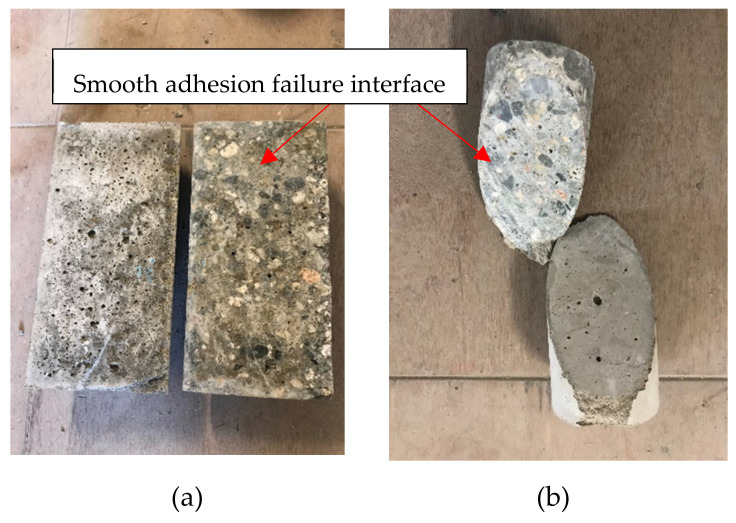
Failure patterns of bond test (**a**) Splitting tensile test (**b**) Slant shear test.

The failure mode can be classified into two types, adhesive failure and cohesive failure. Cohesion failure occurs when either the substrate concrete or repair material fails. When the substrate concrete fails, some substrate materials will be left on the half cylinder of repair materials. Adhesion failure occurs when fracture is at the exact concrete-repair interface leaving a clean and smooth failure interface. Figure 8 shows an example of failure pattern of bond tests. We can observe that all repaired specimens had adhesion failure.

### 3.2. Hysteretic Force-Displacement Response

**Figure 9:**
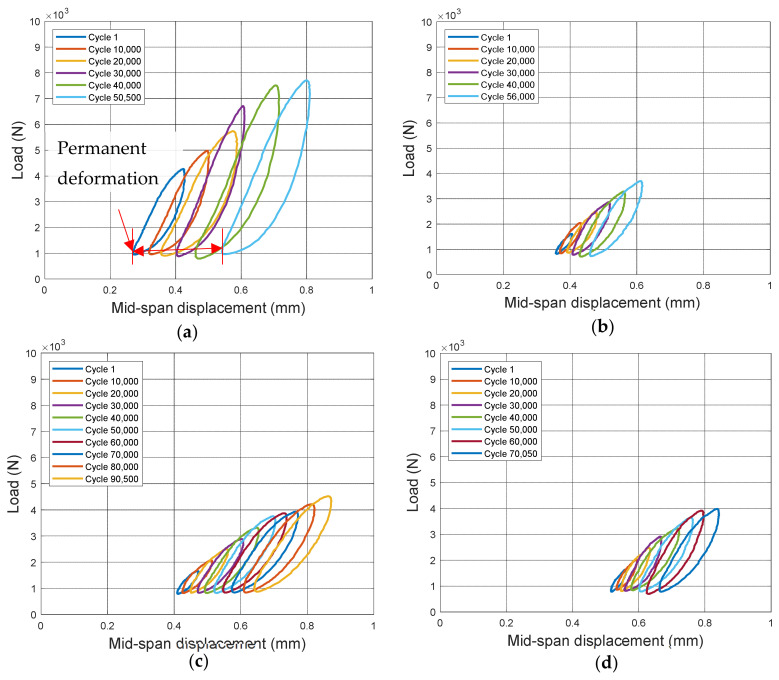
Hysteretic force-displacement curves of representative specimens for (**a**) Mix S (**b**) Mix F (**c**) Mix M (**d**) Mix P.

To simulate in situ-repaired structures, prismatic beams with a square patch on the tension side were prepared and tested under cyclic load. To accelerate the test process, modified loading regime consisting of cycle groups of increasing cyclic stress amplitude is used. The hysteretic force-displacement response of each specimen after each load cycle is recorded, which reflects the damage accumulation and energy absorption capability of the samples [72]. Figure 9 shows the hysteretic curves of representative specimens. Only the results at every 10,000 cycles were plotted for better clarity and readability. A shift in load–displacement curve with increasing number of cycles can be observed for all repair mixes. This shift indicates the permanent deformation after loading cycles, denoted as δ. Table 4 shows the permanent deformation accumulated between the first cycle and failure. Mix F, M, P and S has a δ value of 0.18 mm, 0.22 mm, 0.11 mm, and 0.24 mm, respectively. Disregarding the intact specimens (Mix S), Mix M (cementitious repair mortar) had the highest permanent deformation before failure among all repaired specimens. High inelastic deformation can help delay local failure by redistributing redundant stress in the critical section of a structure and is thus beneficial [73]. Note that Mix M also has the highest fatigue life ($N_{f}$) as shown in Table 4. The superior performance of Mix M repaired specimens to resist cyclic loading could be due to the mechanical characteristics of Mix M. Mix M has an average compressive strength of 68 MPa which is the highest among all the mixes.

**Figure 10:**
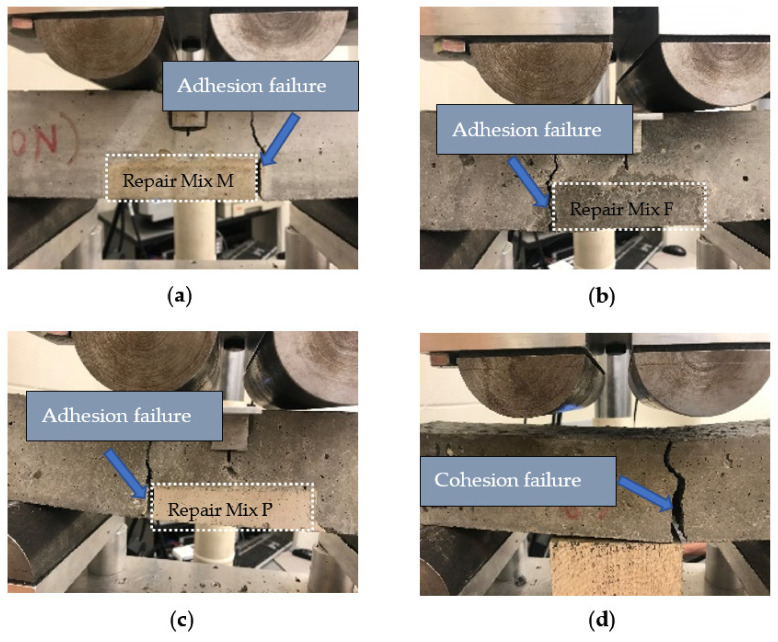
Failure mode under cyclic loading of (**a**) Mix M repaired specimen (**b**) Mix F repaired specimen (**c**) Mix P repaired specimen (**d**) Control Mix S specimen.

Figure 10a–c shows the crack pattern of the repaired specimens under cyclic loading. This failure mode can be classified as adhesion failure [59] which is same as the bond strength test described previously. Adhesion failure occurs when the bond strength between repair and substrate is lower than that of repair and substrate materials. This implies that the chemical bond between the repair and parent concrete is insufficient. Therefore, the use of bonding agent or a rougher bonding interface may be required in this case. Figure 10d shows the crack pattern of the intact specimen (Mix S). This failure mode can be classified as cohesion failure.

### 3.3. Loss in $E_{dyn}$ after Cyclic Loading

**Figure 11:**
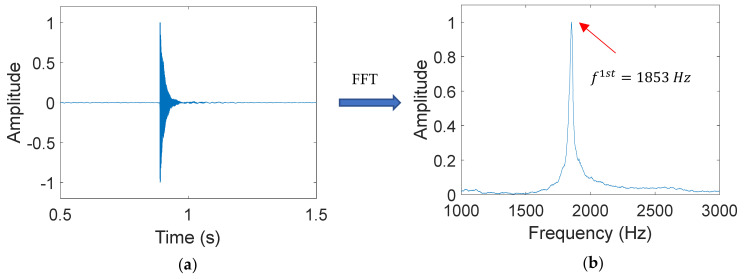
Example of extracting resonant frequency (**a**) Vibration signal generated by hammer impact (**b**) Frequency spectrum obtained via FFT.

Dynamic elastic modulus ($E_{dyn}$) is an important property that indicates the loss in stiffness of a beam caused by cyclic loading. In this study, $E_{dyn}$ is calculated based on the transverse resonant frequency of the 1st mode. Figure 11a shows an example of the vibration signal captured by the accelerometer, and Figure 11b shows the peaks in the frequency domain after fast Fourier transform (FFT) is performed.

**Figure 12:**
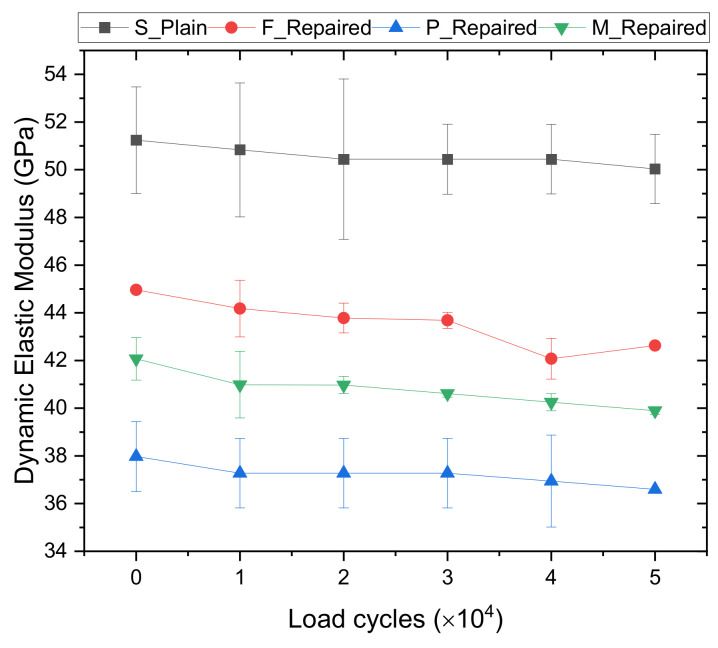
Change in dynamic elastic modulus as a result of flexural cyclic loading.

Figure 12 shows the changes in modulus of elasticity vs. loading cycles. The modulus of elasticity was measured when the maximum cyclic load was 55%, 65%, 75%, 85%, and 95% of the modulus of rupture of each sample type. It can be observed that as expected all mixes experienced a loss during the cyclic loading test. Mix S, F, P, and M had a percentage loss in dynamic elastic modulus of 2.3%, 5.2%, 3.6%, and 5.1%, respectively. Among all the mixes, the intact specimens (Mix S) had the lowest drop in $E_{dyn}$. It is because intact specimens (Mix S) did not have a concrete-repair interface, which contributes to better resistance to cyclic loading compared to repaired samples. Hui-cai et al. [74] proposed a micro-scale model depicting the three-layer structure at the concrete-repair interface. The middle layer contains a great deal of $\mathrm{Ca}{\left(\mathrm{OH}\right)}_{2}$ and needle-shaped Aft crystals, which result in a high porosity and weak bond. Among all the repaired samples, Mix F had the highest drop in elastic modulus (5.2%). This is mainly attributed to the low bond strength of Mix F as summarized in Figure 7.

### 3.4. S-N Curve of Repaired Beams

**Figure 13:**
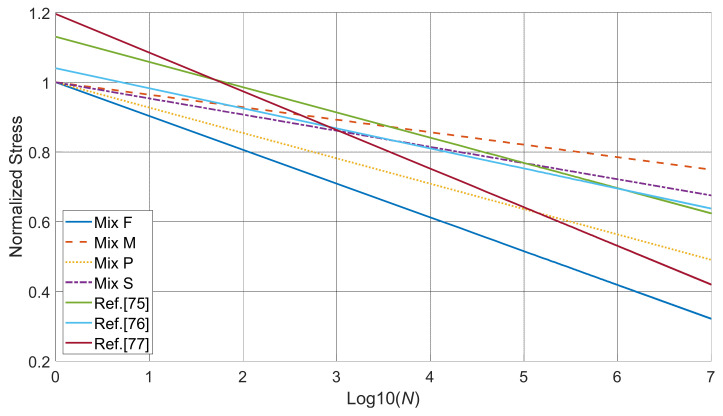
*S-N* curve for different samples.

Figure 13 shows the *S-N* curve for the four repaired mixes. The stress axis in this plot is normalized using the average modulus of rupture results. Based on Palmgren-Miner’s rule, the slope value *b* of Mix S, F, M, and P can be calculated to be −0.033, −0.039.5, −0.013, and −0.026, respectively. In Table 5, *S-N* curves from other researchers [75,76,77] using normal strength concrete are listed for comparison. The slope values in their studies [75,76,77] range from −0.01 to −0.15, and these values from study falls within this range. Note that other studies all used a fixed stress level for each specimen throughout the entire fatigue test process. This study used a novel concept of cycle groups, which accelerates the fatigue test process and can be regarded as producing similar *S-N* curves as other studies [78]. Among all the repair mixes, Mix M showed the lowest absolute slope value in Figure 13 which indicates its superior performance under cyclic loading. This result is validated by the fact that Mix M had the highest average number of cycles before failure (95,991 cycles) compared to other mixes. Specimens repaired with Mix F showed a relatively steep slope, which means the failure stress drops tremendously with the increasing number of cycles. In the case of repaired specimens, the number of cycles is indicative of the bond quality of the repair. The slant shear and splitting tensile tests conducted in this study showed that Mix F had the lowest bond strength regardless of the curing days. Additionally, Mix F repaired specimens showed the largest drop in $E_{dyn}$ during the cyclic loading compared to repair mixes. These results are in line with the predicted *S-N* curve of Mix F.

The flexural fatigue endurance limit is an important design parameter, especially in applications like bridge deck overlays and pavements [78]. Endurance limit is defined as the highest stress the structure can sustain after two-million cycles of non-reversing loading [79,80]. It is believed that if the structure can withstand two-million cycles without failure, it can meet almost all practical purposes [81]. In this study, the fatigue endurance limit of different repaired and control mixes is determined using *S-N* curves. The two-million cycle fatigue endurance limit of Mix F-, Mix M-, Mix P-repaired specimens and the control mix is 38.9%, 77.4%, 54.1%, and 70.8% of the static flexural strength, respectively. The endurance limit of the control mix in this study is compared with the results of normal strength concrete reported in previous literature [78,82,83]. The modulus of rupture value of Mix S in this study is estimated to be 6 MPa based on the building code ACI 318-14 [84]. In a study by Goel and Singh [78], the normal-strength concrete having a modulus of rupture value of approximately 6 MPa was reported to have an endurance limit of 71%, which is similar to the results in this study (70.8%). The flexural strength of the mixes in [82,83] is 5 MPa and 3.28 MPa, respectively, which are lower than that of this study. Accordingly, the endurance limit of the mixes in [82] and [83] was reported to be 58% and 64%, respectively, which is lower than that of this study. Compared with [78,82,83], this study produced similar results with less time consumed indicating the applicability of the novel testing methodology. Mix M shows the highest endurance limit among all repaired specimens including the control mix. However, this does not mean that Mix M-repaired specimens have longer service life than the control mix under fatigue loading as the static flexural strength of the control mix is almost twice as high as that of Mix M-repaired specimens.

## 4. Discussion of Results and Recommendations

In this section, results are supported by general discussion and hypothesis by the authors. Mix S (control mix) showed twice the flexural strength of repaired samples, better energy absorption capacity (higher permanent deformation), and the least drop in $E_{dyn}$ (2.3%) after completing cyclic loading regime. Though Mix S showed the lowest number of cycles to failure (41,311 cycles), it is not the true representation of the resilience of Mix S to cyclic load as Mix S has higher force amplitude applied during cyclic loading as opposed to repaired samples. It is most likely that the concrete-repair interface results in the difference between intact and repaired samples. Among all repair mixes, the *S-N* curve of Mix M showed the least slope which is validated by the number of cyclic load Mix M can withstand, as discussed in the previous section. According to the *S-N* curve, the two-million cycle fatigue endurance limit of Mix M is estimated to be 77.4% of the static flexural strength. To validate this finding, future work will be needed to load the specimen at a value of approximately 77.4% of its static modulus of rupture and determine its fatigue life. As per the Palmgren-Miner rule and Goodman linear model [53], it is assumed that the specimen will fail when the accumulative damage factor $D_{i}$ reaches 100%. As a result, all *S-N* curves in this study in Figure 13 have an intercept of 100%. However, it is seen that the *S-N* in the literature [75,76,77] reported an intercept higher than 100%. It is possible that some samples may not fail even when the maximum cyclic load reaches the static flexural strength of the samples. To address this issue, future work may include introducing a statistical distribution to describe the possibility of the structure failing under cyclic load that reaches 100% of the static load.

## 5. Conclusions

This study explored the applicability of the Palmgren-Miner rule on estimating the fatigue life of repaired concrete structures under cyclic loading. A novel loading regime consisting of increasing cyclic stress amplitude was used to accelerate the test process. The predicted *S-N* curve and 2-million-cycle endurance limit of different repaired specimens were validated by comparing with results including the number of cycles to failure, $E_{dyn}$, slant shear and splitting tensile bond strength, and hysteretic behavior. Additionally, the experimental results using the novel testing regime were compared with those using the traditional fatigue testing method. Based on the results from various tests in this study, the key conclusions are as follows:

1. Mix M (cementitious repair mortar) showed superior bond performance compared with different repair mixes currently used in the field. Mix M had an average shear bond strength of 12.3 MPa and 16.7 MPa and tensile bond strength of 1.4 MPa and 2.3 MPa at 14 and 28 days of curing, respectively. This study confirmed the benefit of using high-strength materials as repair to improve the bond strength.
2. The feasibility of using the Palmgren-Miner rule and Goodman linear model [53] to estimate the fatigue life of repaired structure was confirmed within the context of this study. Mix M, which was estimated to have the highest 2-million-cycle fatigue endurance limit (77.4%), showed the longest fatigue life (95,991 cycles) during cyclic loading test, the highest slant, and splitting bond strength among all repair mixes. Future research may be required to further validate this conclusion by loading the specimen under a fixed cyclic loading range and determining its fatigue life.
3. This study found the usefulness of using cycle groups of increasing cyclic stress amplitude to accelerate the fatigue test process. The two-million cycle fatigue endurance limit estimated using cycle groups of Mix S (70.8%) was very similar to what was reported in the literature (71%) using the traditional cyclic loading method. The use of this method will help shorten the time for performing fatigue tests.
4. The substrate–repair interface was found to have an important role in determining the static and cyclic flexural performance of the repaired structure. Mix S (control mix) showed twice the flexural strength of repaired samples, better energy absorption capability (higher permanent deformation), and the least drop in $E_{dyn}$ (2.3%) after cyclic load. The difference is mainly due to the cold-jointed concrete-repair interface which is the weakest part of the specimens during static and cyclic loading.
5. The predicted *S-N* curve was in line with bond strength, failure pattern, and modulus of elasticity measurements. Specimens repaired with Mix F (cementitious repair concrete), which had a steeper slope compared to other mixes, had the highest drop in elastic modulus (5.2%) and lowest shear and tensile bond strength.
6. The limitation of the Palmgren-Miner rule is that it assumes that the specimen will fail when the damage accumulation reaches 100%, which is contrary to the observations from past literature [78,82,83]. Future research may require the involvement of statistical distribution to account for this phenomenon for improved prediction accuracy.

## Figures and Tables

**Table 1:** Mix design details of the parent material (kg/m^3^) (Data from [42]).

Name	Fine Aggregate	Coarse Aggregate	Cement	Superplasticizer	Air-Entraining Agent	Water
Mix S	762	1053	450	2.25	2.25	153.7

**Table 2:** Details of fresh and hardened properties of substrate and repair materials (Data from [42]).

Material Type	Hardened Properties		Fresh Properties			
	$\mathrm{Density}\;(kg/m^{3})$	$f_{c}^{\prime }\;\mathrm{after}\;28\text{-}\mathrm{Day}\;\mathrm{Curing}\;(\mathrm{MPa})$	Slump (mm)	Time to Set (min)	w/m	Air Content Percentage (%)
Repair Mix M	2325	68±2.4	70	75	0.09	5.0
Repair Mix F	2374	55±2.2	80	-	0.1	5.1
Repair Mix P	2289	39.5±1.3	15	9	0.18	7
Parent Mix S	2530	59.4±1.4	60	90	0.067	4.9

**Table 3:** Loading force groups for different samples.

Percent of Ultimate Load (%)	Mix S		Mix F		Mix M		Mix P	
	Max.	Min.	Max.	Min.	Max.	Min.	Max.	Min.
55	4235	800	2090	800	2090	800	2090	800
65	5005	800	2470	800	2470	800	2470	800
75	5775	800	2850	800	2850	800	2850	800
85	6545	800	3230	800	3230	800	3230	800
95	7315	800	3610	800	3610	800	3610	800
100	7700	800	3800	800	3800	800	3800	800

**Table 4:** Fatigue lives and permanent deformation before failure of different mixes.

Specimen	$\mathrm{Average}\;\mathrm{Permanent}\;\mathrm{Deformation}\;(\delta_{d})\;(\mathrm{mm})$	$\mathrm{Average}\;\mathrm{Number}\;\mathrm{of}\;\mathrm{Cycles}\;\mathrm{to}\;\mathrm{Failure}\;(N_{f})$
Mix F-repaired sample	0.18	58,580
Mix M-repaired sample	0.22	95,991
Mix P-repaired sample	0.11	57,530
Mix S	0.24	41,311

Note: The average results in this table are based on the results of four samples.

**Table 5:** *S-N* curves reported in the literature.

Ref.	*S-N* Curve	Materials
[75]	$S=1.1306-0.0724\log_{10}N$	Self-compacting fiber reinforced concrete containing 0.5% by volume of steel fibers
[76]	$S=1.0401-0.0575\log_{10}N$	Concrete with 0.5% fibers added
[77]	$S=1.1958-0.1109\log_{10}N$	Concrete made with aggregates and 100% unsaturated polyester resin binders

## Data Availability

Some or all data, models, or code that support the findings of this study are available from the corresponding author upon reasonable request.
